# Supplementary material for: Distribution and characteristic of nitrite-dependent anaerobic methane oxidation bacteria by comparative analysis of wastewater treatment plants and agriculture fields in northern China
Source: PeerJ. 2016 Dec 14;4:e2766. doi: 10.7717/peerj.2766 (PMC5160924; doi:10.7717/peerj.2766)
Supplement: Supplemental Information 1 [file peerj-04-2766-s001.docx]

**Distribution and characteristic of** **nitrite-dependent anaerobic methane oxidation bacteria by comparative analysis of** **wastewater treatment plants and agriculture fields in northern China**

**Zhen Hu^a,*^, Ru Ma ^a^**

^a^ School of Environmental Science and Engineering, Shandong University, Jinan, Shandong, China.

Corresponding Author:

Zhen Hu

*No. 27 Shanda South Road, Jinan, Shandong 250100,China*

E-mail: [huzhen885@sdu.edu.cn](mailto:huzhen885@sdu.edu.cn)

**Fig. S1** Rarefaction curves base on pyrosequencing of microbial communities in different samples. The OTUs were defined by 3% distances.

**Fig. S2** Beta diversity analysis of different samples with 2-D PCoA.

**Table S1.** The primers and thermal proﬁles used in the present study.

**Table S2.** Phylotype coverage, diversity and richness estimators of 16S rRNA genes at a phylogenetic distance of 3%.





**Fig. S1** Rarefaction curves base on pyrosequencing of bacterial communities in different samples. The OTUs were defined by 3% distances.

**
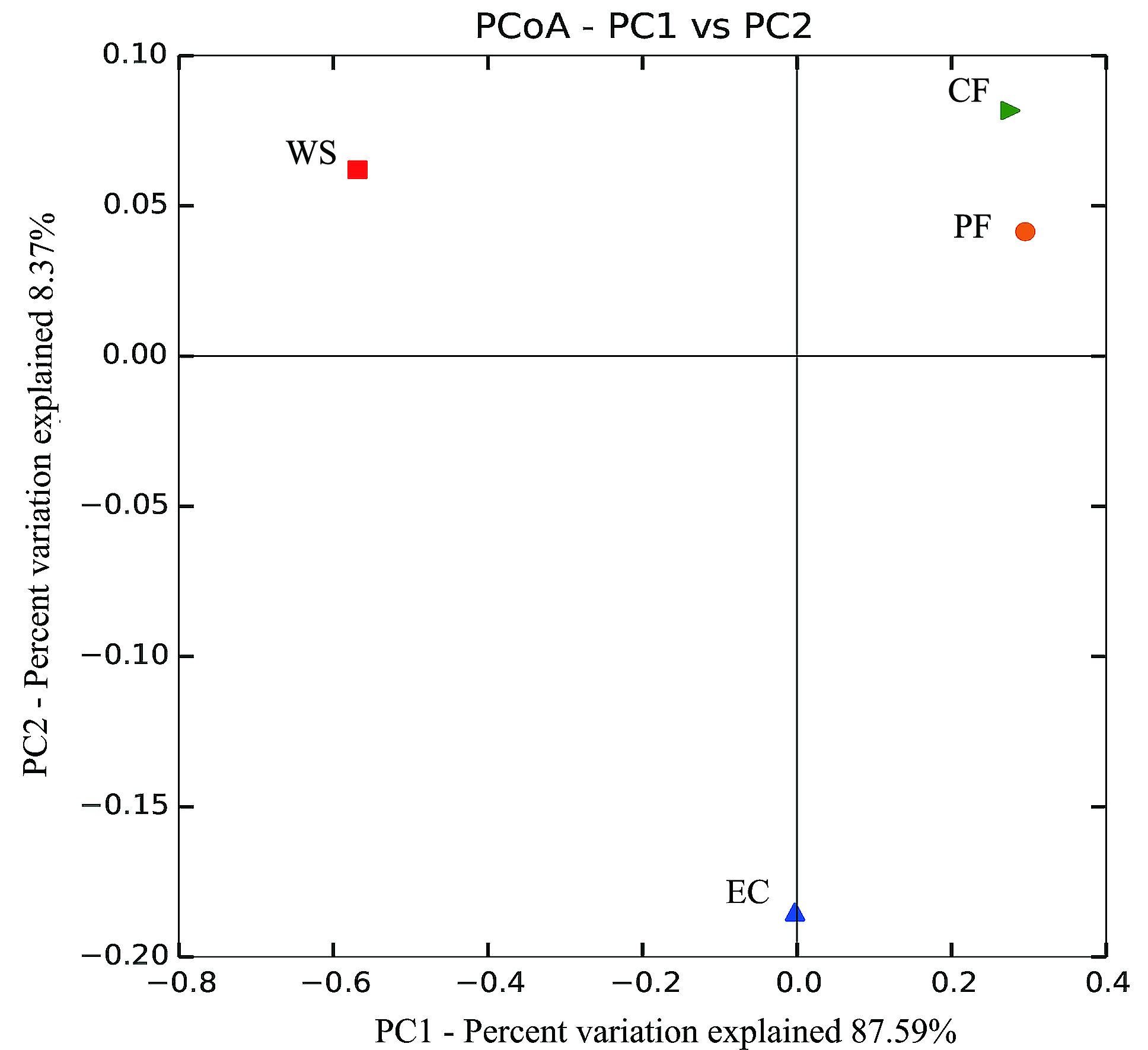
**

**Fig. S2** Beta diversity analysis of different samples with 2-D PCoA.

**Table S1.** The primers and thermal proﬁles used in the present study.

| Primer | Sequence (5’-3’) | Speciﬁcity | Position | Thermal proﬁles | References |
| --- | --- | --- | --- | --- | --- |
| 1545R  202F | CAKAAAGGAGGTGATCC  GACCAAAGGGGGCGAGCG | Bacteria 16S rRNA  *M. oxyfera* 16S rRNA | 1529–1545  193 | 10min at 95℃, followed by 35 cycles of 30s at 95℃, 30s at 51℃and 60s at 72℃ | (GERARD MUYZER 1993; Juretschko et al. 1998) |
| qp1F  qp1R  qp2R | GGGCTTGACATCCCACGAACCTG  CGCCTTCCTCCAGCTTGACGC  CTCAGCGACTTCGAGTACAG | *M. oxyfera* 16S rRNA  *M. oxyfera* 16S rRNA  *M. oxyfera* 16S rRNA | 1001  1201  1481–1500 | 10 min at 95, followed by32 cycles of 60s at 95℃, 60s at 50 ℃and 45s at 72℃(pcr,qp1F- qp2R)  3 min at 95℃, followed by 40 cycles of 60 s at 95 ℃, 60 s at59℃ and 60s at 72℃(qpcr,qp1F-qp1R) | (Ettwig et al. 2009) |

**Table S2.** Raw and effective reads, Good’s coverage, diversity and richness estimators of 16S rRNA genes at a phylogenetic distance of 3%

| Samples | | Raw  reads | Effective reads | OTUs | ACE | Chao | Shannon | Simpson | Good’s coverage (%) | |
| --- | --- | --- | --- | --- | --- | --- | --- | --- | --- | --- |
| PF | 12431 | 10956 | 536 | 972.2 | 1015.3 | 4.11 | 0.81 | 88.13 | |  |
| CF | 11017 | 9528 | 434 | 765.0 | 773.6 | 4.00 | 0.81 | 86.48 |  | |
| EC | 13615 | 12284 | 348 | 601.2 | 580.9 | 3.42 | 0.78 | 90.22 |  | |
| WS | 14814 | 14029 | 203 | 351.7 | 325.2 | 1.30 | 0.26 | 94.70 |  | |
